# Evaluation of Walking Comfort in Children’s School Travel at Street Scale: A Case Study in Tianjin (China)

**DOI:** 10.3390/ijerph181910292

**Published:** 2021-09-29

**Authors:** Jin Zuo, Tong Mu, Tian-Yi Xiao, Jian-Cheng Luo

**Affiliations:** 1 School of Architecture, Tianjin University, Tianjin 300072, China; mutong@tju.edu.cn (T.M.); xiaotianyi@tju.edu.cn (T.-Y.X.); 2 Tianjin Laboratory of Creative Urban Design, Tianjin University, Tianjin 300072, China; 3 Institute of Remote Sensing and Digital Earth, University of Chinese Academy of Sciences, Beijing 100049, China; luojc@radi.ac.cn; 4 Aerospace Information Research Institute, Chinese Academy of Sciences, Beijing 100101, China

**Keywords:** children’s school travel, walking comfort, behavior characteristics, street environment, audit

## Abstract

(1) Background: school travel is an important part of a child’s daily activities. A comfortable walking environment can encourage children to walk to school. The existing methods of evaluating walking environments are not specific to children’s walks to school. (2) Methods: this study proposes a method of evaluating walking comfort in children traveling to school at street scale. Related indexes were selected that reflect children’s school travel behavior and their needs in street environments based on walking environment audit tools. Factor analysis was then used to calculate the relative weight of each index. (3) Results: the new evaluation method was tested in the neighborhoods around the First Central Primary School in Hedong District, Tianjin, China. The walking comfort for children’s school travel was evaluated in eight indexes: effective street width; street flatness; street cleanliness; interface diversity; buffer; shade coverage; green looking ratio; and sound decibels. Different classes and types of streets were found to have various vulnerabilities. (4) Conclusions: this evaluation method can accurately locate the weak spots in streets to improve the local policymakers’ perception of street environments, which can greatly facilitate the implementation of precise measures to promote children walking to school.

## 1. Introduction

Since the United Nations launched the Child-Friendly Cities (C.F.C.) proposal in 1996, 870 cities and regions in the world have obtained C.F.C. certifications, and more than 3000 communities have carried out correlative planning and construction practices. The organization and planning of children’s travel have been paid an increasing amount of attention, and children’s school travel has been an essential aspect. Previous studies have established that walking to and from school has a positive impact on children’s physical and mental health [1,2,3]. Since the streets towards school have undertaken the primary traffic function of children’s walking school travel, they play an essential role in children’s daily activities. An appropriate walking environment for children’s school travel not only stimulates their creativity, imagination, and learning ability, but also shapes their space cognition and promotes social interactions [4]. However, in many middle-income and high-income countries worldwide, rapid urbanization and motorization have reduced children’s walking environmental quality, leading to a significant decrease in the proportion of children walking to school [5,6]. Understanding how to reshape child-friendly street environments has become an urgent issue to be addressed.

Data from several previous studies have demonstrated that the macro levels of urban morphological characteristics, such as land-use mix, residential density, road intersection density, and road network connectivity, are significant influence factors on children’s active school travel [7,8,9]. Some targeted optimization strategies have been proposed, which are difficult to implement by urban planners and managers working in local communities. One potential reason that inhibits the implementation of macro-scale optimization strategies is realistic constraints, such as the shortage of land resources and the high cost of demolition and construction in high-density urban areas. Under the promotion of fine scale urban management, strategies and the means to improve the walking environments of children’s school travel from the micro scale need more attention.

Micro design factors are critical for identifying the quality of walking environments [10], and they have a significant relationship with people’s walking satisfaction [11]. Consequently, we should focus more on walking comfort at micro scale, which means looking for the environmental characteristics affecting children’s perceptions of physical and psychological surroundings. Several studies have proved that walkability indexes, including the length and width of sidewalks [12], street interface [13], shading condition of street trees [14], landscape characteristics [15], public facilities [16], and noise [17] impact the walking experience of pedestrians. Environmental audit tools, such as MAPS [18], PEDS [19], and SPACES [20] are standard measures to quantify microenvironment data and are widely applied in international studies. These tools evaluate the attributes of walking and cycling through professional auditors measuring relevant data on the street. There may be differences in the evaluation indexes selected by audit tools due to differences in pedestrian characteristics (young/old, male/female, fit/unfit), walking purpose (utilitarian/leisure), urban context, social environment, and cultural aspects [21]. However, the importance of the relative weight of influencing factors is ignored by most of those tools. Knuiman et al. examined the relative importance of walkability impact factors. Their longitudinal study in Perth, Australia, showed that walkability elements are not always equally important determinants of walking behavior [22]. The related indexes were weighted mainly through questionnaires [23] and expert scoring [24] in the related studies, where the hierarchical importance of the indexes was estimated according to the preferences of respondents and the professional knowledge of experts. However, both methods have subjective arbitrariness, and significant differences are found between the perspectives of experts and residents on the weight of factors in evaluating walking capacity [25]. There are also some scholars who use objective methods, such as entropy process and factor analysis, to directly use the collected data for calculation, and finally generate index weight values, which have a solid mathematical theoretical basis. These different methods show that the research on index weight is valuable.

Considering the research above, the current investigations are increasingly focused on the impact of built environments on children’s walking commutes to school. However, a key limitation of these studies is that only a few have assessed the impact of environmental factors on children’s school travel at micro scale to guide the design of streets in detail. Another major limitation is that the related literature rarely pays attention to children’s walking comfort requirements on a psychological level during school travel, which requires quantifying children’s subjective feelings. This study aims to propose a method to evaluate walking comfort for children’s school travel at street scale. Children’s subjective feelings and spatial needs for the street environments in school travel were transformed from qualitative expressions into quantifiable indexes. Related environmental data were collected by audit to scientifically and quantitatively evaluate the walking comfort of the street environments. The street network within a 10-min walking distance of the First Central Primary school in the Hedong District of Tianjin was selected as the study site to collect children’s perception of street environment factors and behavior characteristics, aspects which helped to select and add the related indexes in the walking environment audit tools. We have also discussed the results of walking comfort, and analyzed the differences and problems of the different levels and types of streets to provide scientific guidance for the planning, design, implementation, and management of streets.

## 2. Materials and Methods

### 2.1. Framework

**Figure 1:**
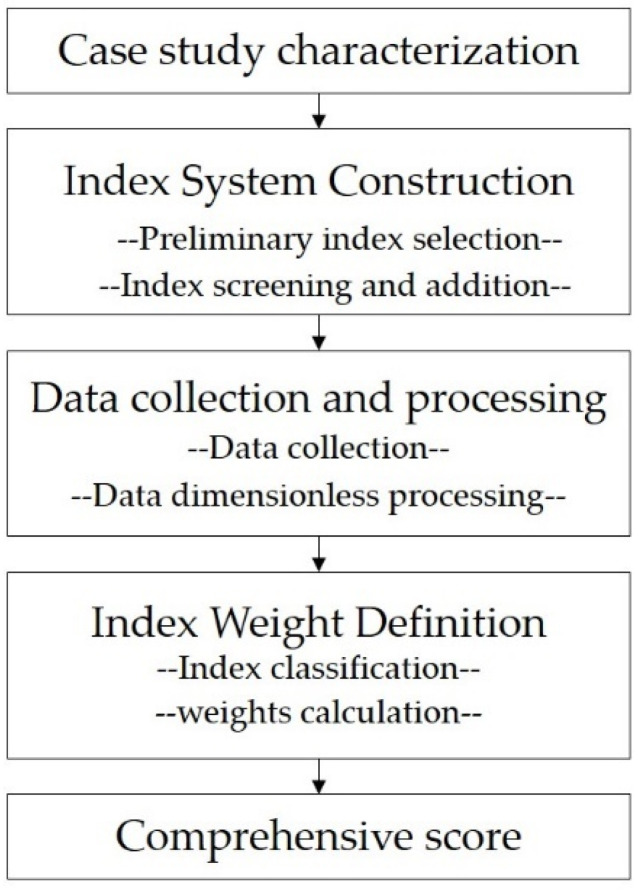
A schematic of the method to evaluate the walking comfort in children’s school travel.

The overall workflow of the walking comfort assessment approach developed for children’s active school travel is shown in Figure 1, in which the following five steps were adopted. First, a representative study area was determined, and geographical scope and scale were defined. Second, the index system was constructed by sorting the microscopic impact factors from the widespread international environmental audit tools, after which indexes were screened, modified, and supplemented accurately according to children’s behaviors and spatial needs. Third, index data were collected through an on-site audit, and calculations and dimensionless quantification were carried out to ensure the accuracy of the data analysis. Fourth, the indexes were classified, and their relative weights were calculated through factor analysis to determine the importance of different indexes. Finally, the walking comfort score was calculated by considering all the indexes comprehensively.

### 2.2. Study Area

As a study area, Hedong District, a typical representation of the central area of Tianjin, was chosen, which has three prominent spatial characteristics, such as (1) an intensive road network with abundant road types, including arterial roads, secondary roads, and branch roads assuming most of the functions of public space in the area, (2) insufficient reserved space for parking development due to the lack of a reasonable prediction of the future parking quantity in its early planning, leading to occupation of the streets, (3) a lack in overall planning of public service facilities, such as seats and bus stations, over different periods, resulting in unreasonable and nonuniform construction in the area.

**Figure 2:**
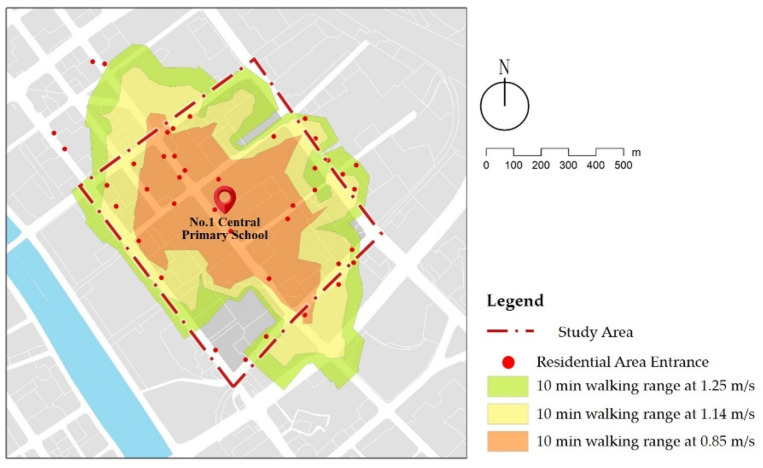
A map of the school service scope and study area.

**Figure 3:**
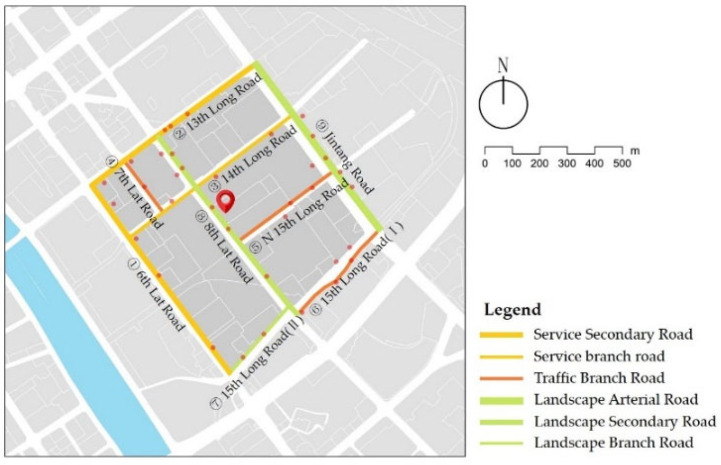
A map of the street classification in the study area.

The principle of nearby primary school enrollment, the Code of Urban Residential Areas Planning and Design (GB50180-2018) [26], requires primary schools to be equipped as public facilities within a 10-min radius of the community living circle. Therefore, children aged six to twelve mainly commute by walking. The average walking speed of children is 1.14 m/s [27]. We selected the First Central Primary school in Hedong District as the study center, and used the GIS network analysis module to calculate the 10-min walking range, which is the service scope of the primary school selected (Figure 2). The streets in the area are divided into arterial roads, secondary roads, and branch roads according to the Standard for Urban Comprehensive Transport System Planning (GB/T 51328-2018) [28]. Accordingly, based on the classification method of street types in the Shanghai Street Design Guidelines [29], the streets are categorized into life streets, traffic streets, and landscaped streets, as shown in detail (Figure 3 and Table 1).

### 2.3. Index System Construction

#### 2.3.1. Audits Overview

This study summarized ten environmental audit tools and reorganized comfort-related indexes into six categories: the street scale (width, slope); street surface (brick paving, flatness, cleanliness); crossing facilities (buffer, safety island, street signs); service facilities (public seats, street lamps, obstacles like the points of drinking water, fire equipment, and trash cans); landscape (street trees); and others (vehicle exhaust, noise, odor, etc.) (Table 2).

#### 2.3.2. Index Modification

**Figure 4:**
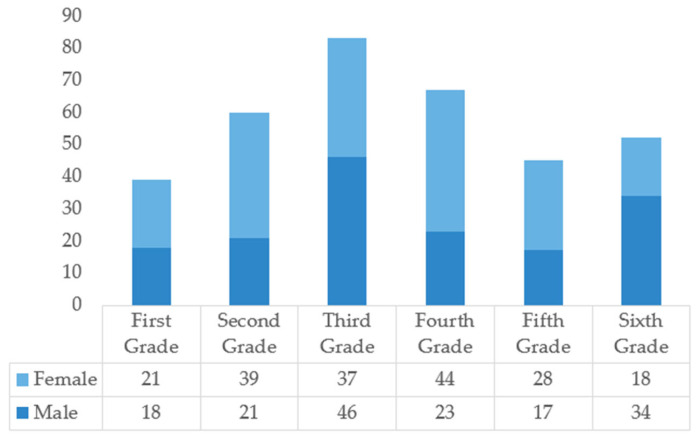
Basic information about the respondents of the study questionnaire.

Field observation, path tracking, and map marking were used to record children’s school travel behaviors and activities. Random interviews with children were conducted after school, and parents and children were invited to complete a questionnaire together. The questionnaire included three parts: children’s basic information; their activities on the street; and their subjective feelings and needs for the street environment. A total of 384 questionnaires were collected, and 346 were valid, with an effective rate of 90.1%. Questionnaire samples were evenly distributed in gender and grade, with high reliability (Figure 4).

**Figure 5:**
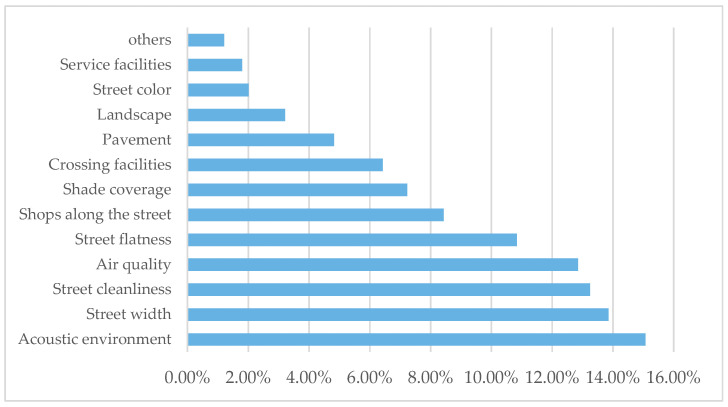
The ranking of children’s perception of environmental factors.

According to the investigation results, the characteristics of children’s school travel are summarized as follows (Table 3, Figure 5):

(1) Time and route: children’s school times are 7:30–8:00 am and 3:30–5:00 pm, which overlap with rush hours. A large flow of people and traffic make it easy for congestion to form, and cause a noisy acoustic environment. Appropriate street width is required for a comfortable acoustic environment. The route to school is relatively fixed. As a result, children are less likely to stop and rest during their school travel, and thus, there is less demand for service facilities such as street lamps, seats, and public toilets.
(2) Typical behaviors going to school: because of the shortage of time, children walk or run, paying less attention to their surroundings, and have a higher demand for road quality. In addition, some children show a state of depression and anxiety when going to school because of the study pressure [37].
(3) Typical behaviors after school: children tend to relax during after-school activities. They walk relatively slowly and are easily attracted to their surroundings. Some children make short stops at the street stalls and convenience stores on the way home. Sometimes, they also walk around the 10 min life service circle of the school to complete daily activities, such as shopping, leisurely activities, and eating with their parents [38], so the demand for a suitable environment interface is high. Jan Gehl proposed that, compared with the aspect ratio and height difference of the street interface, the diversity of the street bottom interface has more influence on street activities [39].

According to the above-mentioned behavioral characteristics of children walking to school, we found that children have a higher demand for road quality and interface diversity than service facilities, because of their clear destination. This study selected effective street width, street flatness, street cleanliness, and interface diversity to measure children’s walking comfort. According to children’s psychological characteristics, exposure to the natural environment can improve emotional health [40]. In addition, a study found that people’s psychological recognition of street greening with rich levels will offset some of the results of a low actual comfort evaluation index [41]. One index to measure the quality of a street’s natural environment is the green looking ratio (see Table 4), which is the proportion of green plants in objects seen by people’s eyes, emphasizing subjective visual effects [42].

In addition, climate comfort is one of the aspects that influence human feelings. The literature demonstrates that microclimates (including air environment, thermal environment, and wind environment) impact the comfort of pedestrian walking [43]. Vehicle exhaust is a primary factor influencing the air environment. Urban vegetation can improve air quality by affecting the deposition and diffusion of pollutants, so the placement of vegetation that is close to the surface and the source of pollution, to dilute emissions, is essential [44]. Therefore, setting up green belts is one of the currently adopted road ecological prevention and control measures to reduce the diffusion of road pollutants. Street trees are shown to effectively adjust the effects of the urban heat island effect [45] and the urban street canyon effect [46] through transpiration, evapotranspiration, shading, and reflection. Consequently, the form of buffer (either with or without shrubs, or other low plants between motorways and sidewalks) was examined when observing street air quality, and the shade coverage of street trees was used to monitor thermal and wind conditions.

Consequently, we determined eight indexes and described their definition: data measurement and calculation methods, including effective street width ($X_{1}$); street flatness ($X_{2}$); street cleanliness ($X_{3}$); interface diversity ($X_{4}$); buffer ($X_{5}$); shade coverage ($X_{6}$); green looking ratio ($X_{7}$); and sound decibels ($X_{8}$), all of which are important to children’s comfort when walking on streets (Table 4).

### 2.4. Data Collection and Processing

#### 2.4.1. Data Collection

We obtained road network data and land use spatial layout data in the study area through OpenStreetMap (https://www.openstreetmap.org/ (accessed on 5 April 2021)), and acquired the POI (point of interest)to attribute the assignment of spatial data using Baidu Map API, including building functions, facility names, and road names. We used ArcGIS to effectively integrate multi-source data, facilitating data reading, editing, and visual presentation as the basic spatial data of this study.

**Figure 6:**
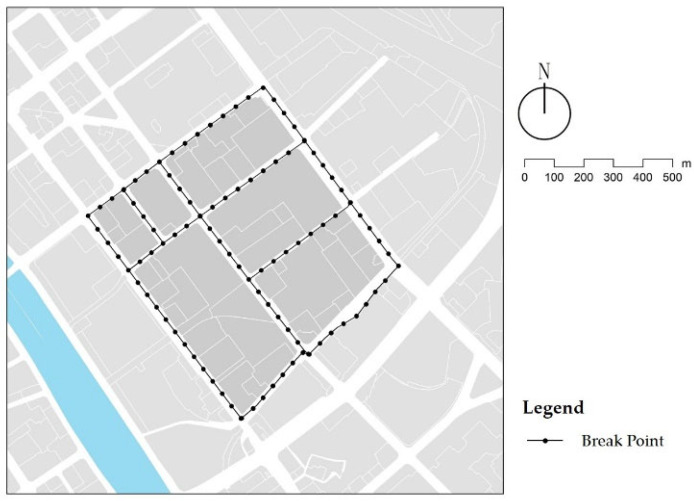
Sampling site in study area.

The index data were collected by a site survey and remote sensing images. The elements of streets and street trees were extracted from high-resolution remote sensing images to calculate the street area and projected canopy area of street trees. The data collection requirements of the site survey included the selection of the street on the side with more entrances and exits to the residential area, or with more pedestrian volume during school travel time, as the research object. We considered 50 m as the basic unit for segmentation and as the identifier. In cases where the length of the road section was less than 25 m, it was merged with the previous section of the road, corresponding to 102 street units (Figure 6). Auditors recorded the relevant data of indexes at each street unit, including quantitative data, qualitative data, and street view photos. Quantitative data measured specific values, including the effective street width, the number of types of street interface, and the sound decibel value. Qualitative data referred to the data described by state and degree, including street flatness, street cleanliness, and plant barriers. Street view photos were taken from a child’s perspective (1.1 m height) with the street as the visual center. We trained the auditors using a standard protocol to give them unified measurement criteria and judgment standards to minimize the reporting bias between different interviewers. Auditors collected these data many times when children commuted to school and home, compared the data, and filtered the outliers to ensure the reliability and accuracy of the data.

#### 2.4.2. Dimensionless Data Processing

Different types of indexes cannot be evaluated comprehensively because qualitative and quantitative indexes are collected differently, and have different units. For example, street flatness and street cleanliness are qualitative evaluation factors rated from 1 to 3, whereas the buffer is a qualitative evaluation factor classified as 0 or 1 based on whether a green belt exists between motor lanes and non-motor lanes. In addition, the effective street width, the interface diversity, the shade coverage, the green vision rate, and the acoustic environment are quantitative evaluation factors calculated using different software to the actual measured values. Because of the different attributes of indexes, there may be a bias in a direct calculation of the comprehensive score with the original data of indexes. The larger the magnitude difference of the index variables, the greater the impact of the index on the comprehensive score. Therefore, we conducted dimensionless processing on the actual data of indexes, mapping the actual data of each index to a standard and appropriate interval to ensure the comparability of the index data [47]. We used the zero-mean normalization method (Z-score) to transform the actual data to calculate the relative weight of indexes and comprehensive evaluation analysis using the formula:

(1) $$\{\begin{array}{cc} & y_{i}=\frac{x_{i}-\bar{x}}{s}\\ & \bar{x}=\frac{1}{n}\overset{n}{\underset{i=1}{\sum }}x_{i}\\ s= & \sqrt{\frac{1}{n-1}\overset{n}{\underset{i=1}{\sum }}{\left(x_{i}-\overset{¯}{x}\right)}^{2}} \end{array}$$

where $y_{i}$ is the evaluation data, $x_{i}$ is the measured data, $\bar{x}\;$ is the average value of the measured data, and s is the variance of the measured data.

### 2.5. Index Weight Definition

To avoid the weight bias caused by the correlation between evaluation indicators, we conducted a factor analysis on the measurement data of eight indexes, and the objective weight of each indicator was calculated (Table 5).

### 2.6. Comprehensive Score Determination

We calculated the comprehensive quantitative score V of walking comfort of children in each street to clarify the direction for improvement using the formula:

(2) $$V=\sum_{i=1}^{n}\alpha_{i}X_{i}$$

where $\alpha_{i}$ is the weight of the evaluation index, and $X_{i}$ is the score of the evaluation index.

## 3. Results

The audit results of each index were categorized according to the relevant standards or through the natural breaks into three levels, “discomfort”, “general”, and “comfort”, and scored between 1–3 points. According to the specifications of the Standard for Urban Comprehensive Transport System Planning (GB/T 51328-2018) [28], the minimum street width must not be less than 2 m; hence, we divided effective street width ($X_{1}$) into three levels: $X_{1}$ ≤ 1.5 m, 1.5 m < $X_{1}$ < 3 m, $X_{1}$ ≥ 3 m. Referring to the Shanghai Avenue Construction Guidelines, street shade coverage should reach more than 90%. As such, shade coverage ($X_{6}$) was divided into: $X_{6}$ < 50%, 50% ≤ $X_{6}$ < 90%, $X_{6}$ ≥ 90%. The Standard for the Environment Noise defines that the environmental noise limit of residential, cultural, and educational areas is 55 dB in the daytime and 45 dB at night. Thus, the sound decibels ($X_{8}$) were divided into three levels: $X_{8}\leq 45\;\mathrm{dB}$, $45\;\mathrm{dB}<X_{8}\leq 55\;\mathrm{dB}$, $X_{8}>55\;\mathrm{dB}$. The interface diversity ($X_{5}$), shade coverage ($X_{6}$), and green looking ratio ($X_{7}$) were graded by the natural breaks method (Table 6). Each index was further analyzed to identify weaknesses in each street, providing detailed insights into possible problems identified. Finally, the comprehensive score of each street was calculated to make an evaluation of walking comfort for children’s school travel.

### 3.1. Effective Street Width

**Figure 7:**
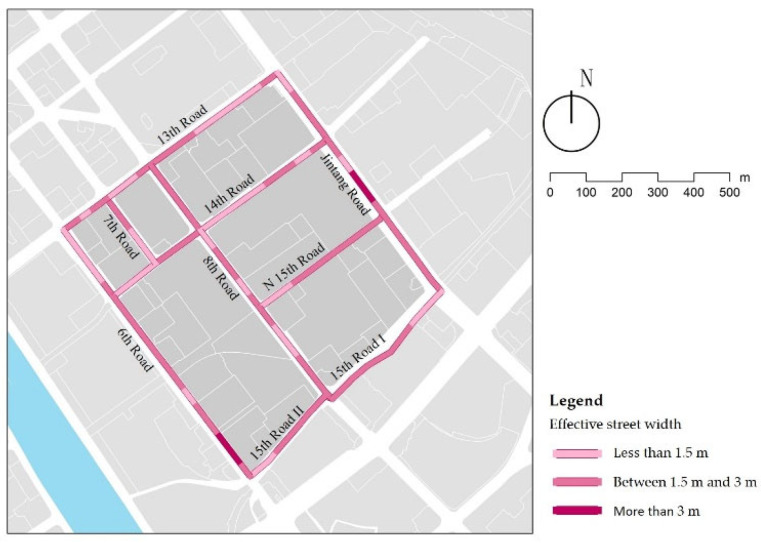
Examples of effective street width.

**Figure 8:**
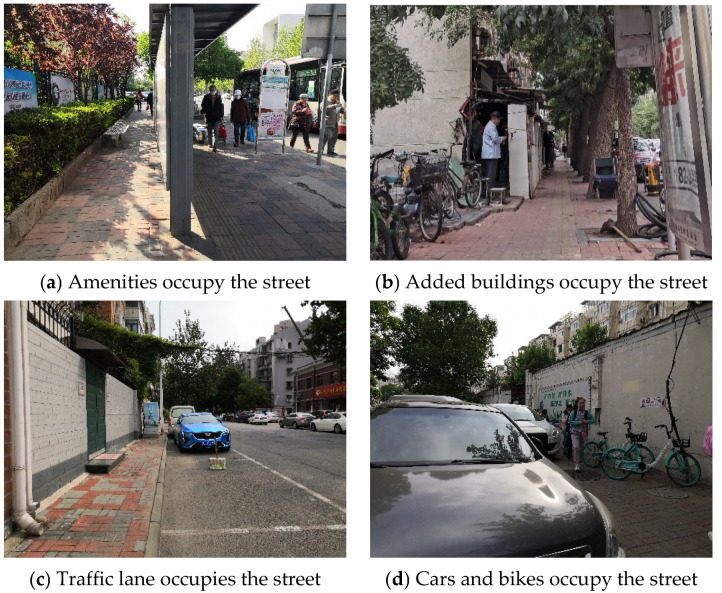
Reasons for street occupancy.

According to the road construction requirements, the width of street construction varies for different road classes, and it is recommended that arterial roads be 3–7 m, secondary roads 3–6.5 m, and branch roads 2–5 m. Table 7 records the actual construction width and effective width of each street. The actual construction width of the streets basically meet the specification requirements, but 33% of the streets are less than 1.5 m, which reduces walking comfort in children’s school travel (Figure 7). Though secondary streets and branch streets assume part of the functions of public space, some street public services, such as seats and bus stops, are poorly laid out, greatly reducing the effective street width (Figure 8a). Further, building additions and motorway extensions have further compressed the street width (Figure 8b,c). In addition, street sections with a high density of shops along the street and street sections at the entrances and exits of residential areas are commonly temporarily occupied by vehicles and bike-sharing, which are major obstacles on the street, resulting in an effective street width of less than 1.5 m or even 0 m, which has a negative impact on children’s walking comfort (Figure 8d). In summary, the effective street width is influenced by road hierarchy, placement of facilities, and users. It needs to be addressed in subsequent street design and management, through the proper planning of public facilities and real-time parking management measures.

### 3.2. Street Flatness

**Figure 9:**
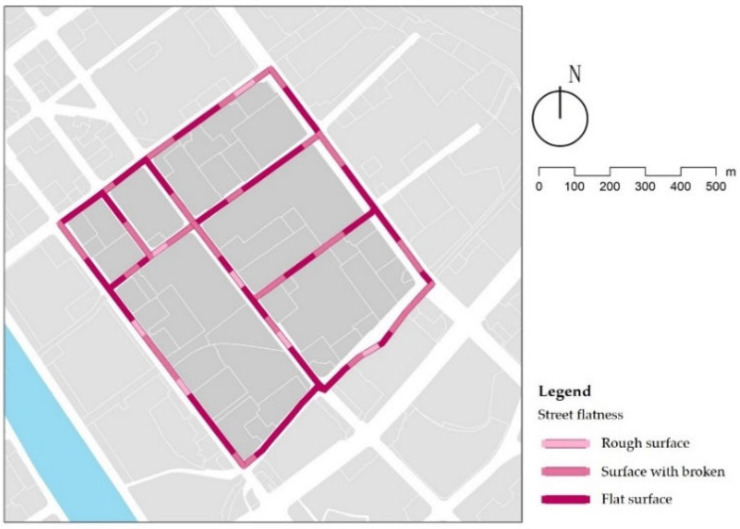
Examples of street flatness.

**Figure 10:**
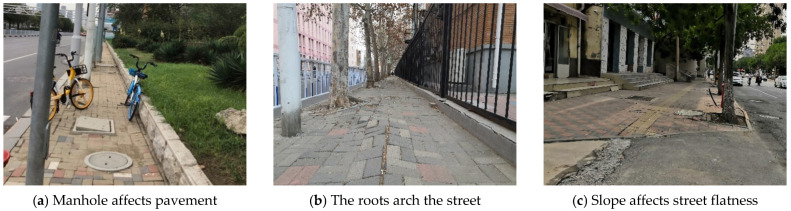
Reasons for uneven street surface.

Over 56% of the streets show a level of comfort (Figure 9). However, 7% of the streets have a quality of pavement that can be very disruptive to children walking, due to uneven paving blocks caused by a poor fit between well covers and paving (Figure 10a) and undulations in the pavement due to tree root growth arching the surrounding pavement (Figure 10b). In addition, 37% of the streets have slightly uneven surfaces, mainly found at street intersections with residential entrances. Street designs usually eliminate the curb height difference, which creates a slope that causes the brick paving not to fit (Figure 10c). In street renewal, repair and reconstruction of paving that affect pedestrian is a priority, and in long-term street planning and design, existing hard paving should be replaced with permeable asphalt pavement to avoid buckling and missing paving blocks.

### 3.3. Street Cleanliness

**Figure 11:**
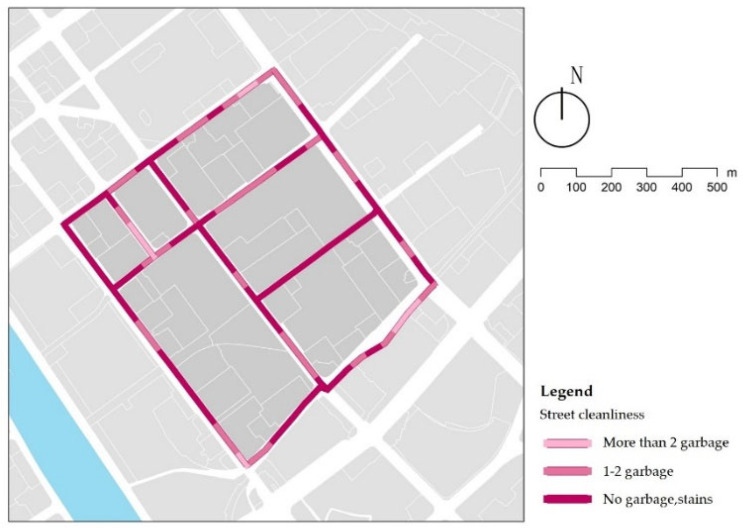
Examples of street cleanliness.

**Figure 12:**
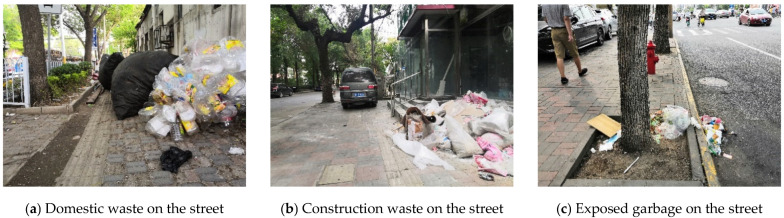
Examples of street uncleanliness.

The results show that 61% of the streets are clean, and only 6% have poor sanitation (Figure 11). It is mainly concentrated in the stacking of domestic waste on the street at the waste transfer station on 7th Lat Road (Figure 12a), and construction waste on 13th Long Road (Figure 12b). Except for the sections with poor cleanliness, it was found that the overall cleanliness of the streets is better on landscape streets than traffic streets and service streets (Table 8). This phenomenon can be explained by the combination of many shops along the service streets and a small amount of garbage cans. As such, garbage is exposed in certain sections of the street (Figure 12c). In street cleaning work, the garbage can layout and street-cleaning frequency can be arranged according to the functional type of the street.

### 3.4. Interface Diversity

**Figure 13:**
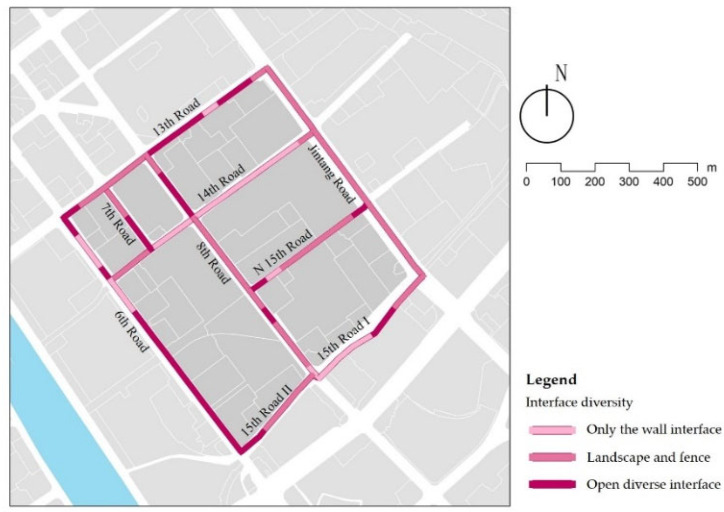
Examples of interface diversity.

The street interfaces can be summarized as open spaces, shops, landscapes, fences, and walls. The types of interfaces were counted for each street, and the results are as follows: 31% of the street interfaces are in the comfort category, concentrated in the service streets, indicating that most children’s shopping and playing behaviors concentrate here (Figure 13); and 21% of the street interfaces are relatively homogeneous, like 14th Long Road, whose interface is primarily walls, forming a boring pedestrian atmosphere. The data show (Table 9) that the interface diversity is related to the street types, with service streets having the most interface types. As mentioned earlier, secondary roads and branch roads carry most of the children’s activities. Their interfaces should be designed to match children’s activities by combining architectural function with landscape sketches, e.g., replacing walls with plants or fences and encouraging children’s participation in wall painting to weaken monotonous street environments through color.

### 3.5. Buffer

**Figure 14:**
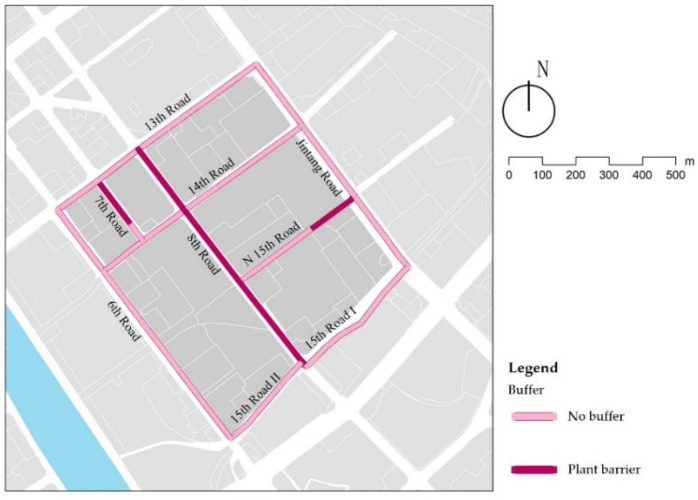
Examples of buffers.

**Figure 15:**
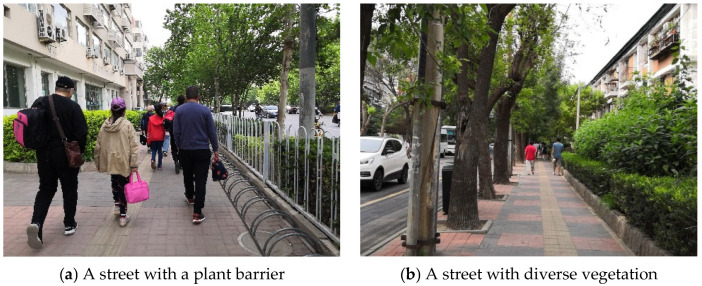
Photos of streets with different kinds of vegetation.

Only 8th Lat Road has a plant barrier among the secondary roads, and among the branch roads, 7th Lat Road and part of North 15th Long Road have a plant barrier (Figure 14). The height of shrubs is close to the average height of children (1.1 m), effectively reducing the negative impact of vehicle exhaust on children’s school travel (Figure 15a). Although diverse vegetation is planted along Jintang Road and 15th Long Road, it does not serve as a barrier to the dispersion of pollutants, such as vehicle emissions (Figure 15b). According to the significant difference in the traffic flow of different roads in Tianjin (arterial roads (68 vehicles/min) > secondary roads (41 vehicles/min) > branch roads (25 vehicles/min)), a plant barrier should be constructed on arterial roads and secondary roads to reduce the impact of vehicle exhaust on children’s school travel.

### 3.6. Shade Coverage

**Figure 16:**
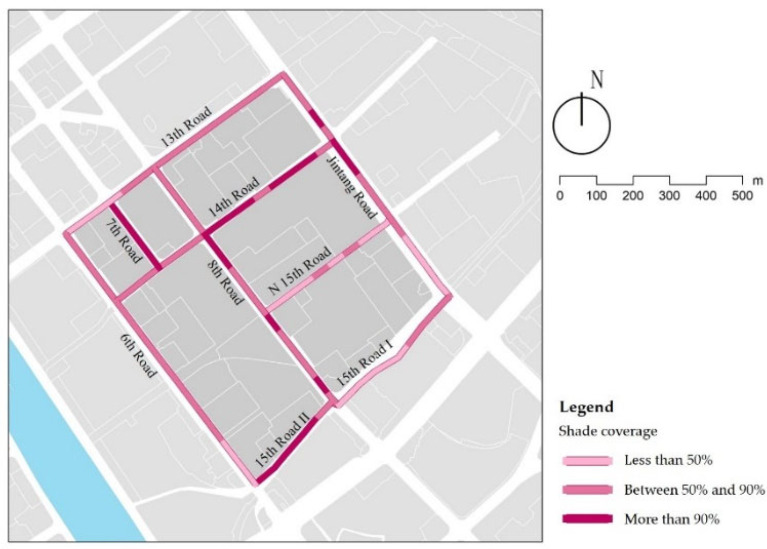
Examples of shade coverage.

Only 21% of the streets have 90% shade coverage, mainly in the branch roads (Figure 16). Because street trees planted with appropriate densities in the branch roads are older and have a larger canopy radius, most street sections achieve full shade coverage. The streets with lower than 90% shade coverage have so primarily due to discontinuous shade with a low density of street trees, as is the case on 6th Lat Road, or a relatively small canopy radius because of different tree species, as is the case on 8th Lat Road. Some street sections have no street trees, thus lacking shade and reducing walking comfort, as is the case with Jintang Road (Table 10). This can be improved by increasing the density of trees and adjusting the species.

### 3.7. Green Looking Ratio

**Figure 17:**
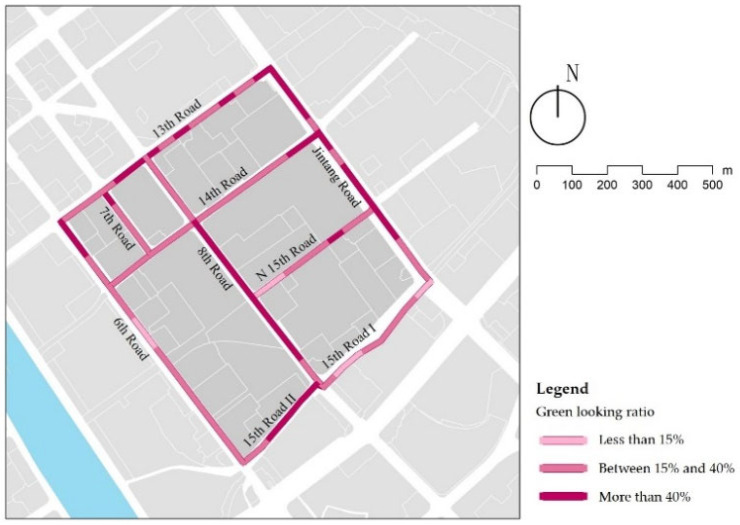
Examples of green looking ratios.

One study found that people perceived the streets as more artificial when there was less than 15% greenness [48]. The results show that only 6% of the streets in the study area have a green looking ratio lower than 15%, mainly in the street sections without street trees (Figure 17). Excluding them, each street’s average green looking ratio was calculated (Table 11), and it was found that the landscape streets are the best, due to a variety of shrubs and plants along the street, and as such, the amount of green is sufficient, and children can easily see the plants. There is not much difference between the green looking ratio of service streets and traffic streets, and both are mainly affected by street trees and vertical greening (such as ivy). The amount of grass, shrubs, and other harmless plants should be considered in street design.

### 3.8. Sound Decibels

**Figure 18:**
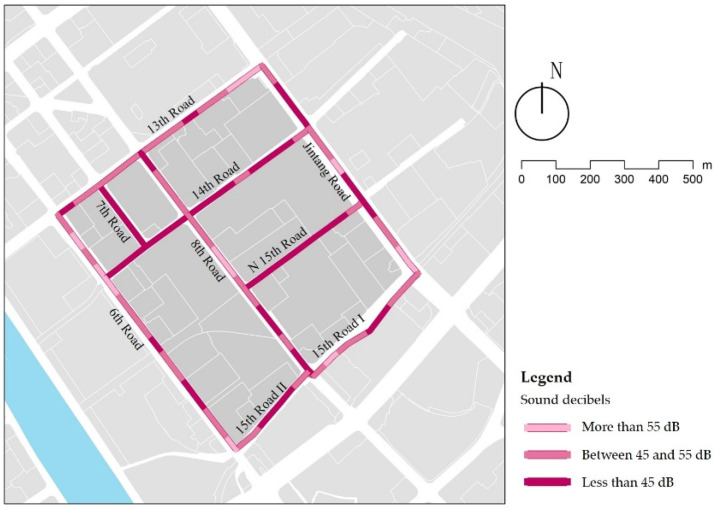
Examples of green sound decibels.

Traffic noise and life noise are the primary noise sources in streets. The results show that the noise of the branch roads is mostly lower than 45 dB, significantly better than the other streets (Figure 18), because of smaller traffic flow and pedestrian flow during the children’s school travel time. Of all the streets examined, 12% of them had noise exceeding 55 dB. Part of this is due to the street around the primary school, where the congestion is easy to form with the increase of pedestrian and vehicle density, and the car horn sounds have a noise level of more than 55 dB. Other street sections have noise levels over 55 dB from nearby construction. In addition, the audit found that birdsongs have a positive effect on improving the acoustic environment, diverting children’s attention from the sound of car horns. In street design, a street soundscape should be created to enhance the comfort of the street sound environment. In street management, it is possible to reduce the traffic noise by limiting the speed and indicating no honking in some sections.

### 3.9. Comprehensive Evaluation

**Figure 19:**
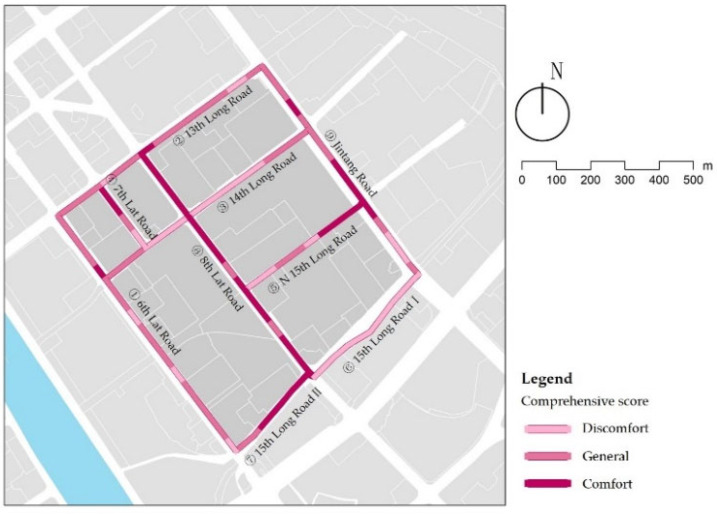
Results of comprehensive evaluation.

After standardizing the data, the indexes were superimposed based on their weights to calculate a comprehensive score of walking comfort in children’s school travel on each street (Table 12). Using natural breaks method, the results were categorized into three levels: “discomfort”; “general”; and “comfort”. The results in the study area indicate apparent pluralism in the comprehensive evaluation of different street types (Figure 19). Integration of the evaluation data shows that in terms of street types, the landscape streets are the best, followed by the service streets, and the traffic streets are the worst. In terms of street hierarchy, the arterial roads and secondary roads are more comfortable than branch roads (Table 13). It is essential to locate the weak points in the streets precisely, and to implement delicacy management.

Based on the previous analysis of individual indicators, street cleanliness, interface diversity, and the green looking ratio vary significantly by street type, and different street hierarchies have significant differences in forest coverage and sound decibels. All streets have problems with encroachment by vehicles, bike-sharing, public facilities, and uneven pavement due to well covers, tree roots, and slopes, which significantly impact children’s walking comfort. We found that the landscape streets have comfortable cleanliness and green views, but their interfaces are homogeneous. The function compound along the street needs to be enhanced to form an active space. The service streets have poor street cleanliness, and some public facilities such as garbage cans can be laid out depending on the density of shops along the streets. The traffic streets in the study area all belong to the branch roads, where a single interface and low green views are the main problems. Replacing part of the residential wall with landscape design can increase the interface diversity and the interaction between children and nature. The tree species selection and planting density of street trees need to be improved for the secondary streets. In addition, children are more affected by polluting gases, such as vehicle exhaust and street dust, due to their height limitations. It is essential to set plant barriers between streets and motorways to create a comfortable and natural walking environment for children’s school travel.

## 4. Conclusions

Children’s physical characteristics and behavior activities are different from those of adults, so children’s needs for walking comfort are different in the same built environments. School travel as a compulsory behavior, with its characteristics of concentrated time and fixed routes, is an important part of daily life that affects children. Research has proven that micro-design factors of built environments strongly influence the walking comfort in children’s school travel. Effective assessment of the street environment is a prerequisite for optimizing school travel roads. This study explored the characteristics of time, space, and behavior of children’s school travel, and their needs in their walking environments. It proposed a method of evaluating walking comfort in children traveling to school at street scale, including index selection, data collection, and data analysis. This study audited each environment factor in the street network within a 10-min walking distance of a primary school, and used GIS to analyze objectively measured data in order to locate weaknesses in the streets and improve the local policymakers’ perception of street environments, which can greatly facilitate the implementation of precise measures to promote children walking to school.

The innovation of this study is to transform children’s subjective feelings and qualitative expressions in school travel into quantifiable indexes. In addition, compared with commonly used audit tools, this study considered the relative importance of each index in children’s school travel, and used factor analysis to objectively assign weights to the indexes to more realistically reflect the evaluation results of the street environment. This study has several limitations, however. First, further research is needed on the possible effects of parental perceptions, and their attendance, on children’s behavior in school travel. Second, this study focused on quantifying subjective perceptions of children’s walking comfort, and did not consider factors influencing children’s safety and street accessibility (such as crossing signals and effective street length). Therefore, future research needs to consider parents’ and children’s perceptions of street environments, and a collaborative study should be conducted on objective and subjective environmental factors, such as safety, accessibility, and comfort in children’s school travel.

## Figures and Tables

**Table 1:** Street classification in the study area.

Num.	Street Name	Street Hierarchy	Street Type	Street Characteristics
1	6th Lat Road	Secondary road	Service street	A street dominated by service-oriented commercial, retail, catering, and public facilities that serve local residents.
2	13th Long Road	Secondary road	Service street	
3	14th Long Road	Branch road	Traffic street	A street with a non-open interface and vital traffic function.
4	7th Lat Road	Branch road	Traffic street	
5	North 15th Long Road	Branch road	Traffic street	
6	15th Long Road I	Branch road	Traffic street	
7	15th Long Road II	Branch road	Landscape street	A street with prominent landscape features, allowing concentrated leisure activities facilities.
8	8th Lat Road	Secondary road	Landscape street	
9	Jintang Road	Arterial road	Landscape street	

**Table 2:** Environmental audit tools and related indexes.

Name of Tools	Researchers	Rough Categorization of Indexes													
		Street Scale		Street Surfaces			Service Facilities			Crossing Facilities			Landscape		Others
		Width	Slope	Paving	Flatness	Cleanliness	Seats	Lamps	Obstacles	Buffers	Refuge island	Signs	Trees	Landscape	
Pedestrian Environment Review Software(PERS)	TfL, TRL, * (2001) [30]	**·**	**·**		**·**			**·**			**·**			**·**	
Systematic Pedestrian and Cycling Environmental Scan(SPACES)	Pikora et al. (2002) [20]	**·**				**·**		**·**	**·**				**·**		**·**
Walking Suitability Assessment Form (WSAF)	Emery et al. (2003) [31]	**·**	**·**	**·**				**·**		**·**					
Path Environment Audit Tool (PEAT)	Troped et al. (2006) [32]		**·**		**·**		**·**	**·**	**·**	**·**	**·**	**·**			**·**
Irvine Minnesota Inventory (IMI)	Boarnet et al. (2006) [33]	**·**	**·**				**·**	**·**				**·**	**·**		
Scottish Walkability Assessment Tool (SWAT)	Catherine et al. (2008) [34]			**·**						**·**				**·**	
PIN3 Neighborhood Audit Instrument (PIN3)	Evenson et al. (2009) [35]	**·**	**·**		**·**			**·**		**·**		**·**	**·**		
Pedestrian Environmental Data Scan (PEDS)	Clifton et al. (2009) [19]		**·**	**·**	**·**	**·**		**·**	**·**	**·**		**·**	**·**		
Microscale Audit of Pedestrian Streetscapes (MAPS)	James F. et al. (2015) [18]				**·**		**·**	**·**	**·**	**·**			**·**		**·**
Walkability Explorer(WE)	Ivan et al. (2015) [36]	**·**	**·**	**·**			**·**	**·**		**·**					**·**

* TfL stands for Transport for London, TRL stands for UK’s Transport Research Laboratory.

**Table 3:** General characteristics of school-age children.

	Time	Psychological State	Behavior	Attention	Stops	Space Needs
Go to school	7:30–8:00	Depression, anxiety	Walk and run faster	Paying less attention to surroundings	Breakfast shops, stalls	Flat streets with appropriate width, comfortable landscape
Go home	15:30–17:00	Relaxed, excited	Walk and run slower, stay	Easily attracted to surroundings	Markets, restaurants, stores	Diversified interface, clean streets, comfortable landscape

**Table 4:** Walking comfort index of children’s school travel.

Index	Definition	Data Measurement	Calculation Method
Effective street width ($X_{1}$)	The actual walking width of a street.	The number of unoccupied bricks in a row of paving on the street (20 cm/p) was counted.	$X_{1}=B_{s}-B_{o}$ $B_{s}$, the street width, $B_{o}$, the width occupied by amenities.
Street flatness ($X_{2}$)	The deviation value of longitudinal bump of pavement.	Qualitative scores were given on the impact of walking according to the damage degree of paving.	Rough surface: $X_{2}$ = 1; Surface with a bit of broken: $X_{2}$ = 2; Flat surface: $X_{2}$ = 3.
Street cleanliness ($X_{3}$)	The amount of dirt left on the pavement after cleaning.	Qualitative scores are conducted for Requirements for Quality and Operation of City Road Sweeping and Cleaning (DB11/T353-2014).	More than 2 garbage, stains: $X_{3}$ = 1; 1–2 garbage, stains: $X_{3}$ = 2; No garbage, stains: $X_{3}$ = 3.
Interface diversity ($X_{4}$)	The number of building facade types and element types (such as walls, shops, fences, plants).	The main types of spatial elements included in the interface of each street unit were recorded by site survey.	$X_{4}=\frac{N}{L_{s}}$ N, the number of interface types contained in the street unit, $\;L_{s}$, the length of the street unit.
Buffer ($X_{5}$)	The plant barriers between the vehicle road and the street.	The presence or absence of plant barriers was recorded by site survey.	No buffer: $X_{5}$ = 0; Plant barrier: $X_{5}$ = 1.
Shade coverage ($X_{6}$)	The percentage of streets covered with tree shade.	The elements of streets and street trees were extracted from high-resolution remote sensing images and their areas were calculated by GIS.	$X_{6}=\frac{S_{t}}{S_{s}}$ $S_{t}$, the area of the street covered with trees, $S_{s}$, the street area.
Green looking ratio ($X_{7}$)	The proportion of plants in objects seen on the street from a child’s perspective.	Streetscape photos were taken from a child’s perspective (1.1 m height) on the street, and the area ratio of plants in the photos was calculated through image segmentation.	$X_{7}=\frac{S_{g}}{S_{z}}$ $S_{g}$, the area of green plants in the street view image, $S_{z}$, the area of street view image.
Sound decibels ($X_{8}$)	The intensity of sound in the street during the school travel time.	The decibel value of each street unit was measured by the Noise Decibel Meter Application.	$X_{8}=$ Decibels measured

**Table 5:** The weight of each index.

Index	Weight
Effective street width	0.138
Interfacial diversity	0.122
Street flatness	0.101
Street cleanliness	0.113
Shade coverage	0.158
Green looking ratio	0.144
Buffer	0.126
Sound decibels	0.098

**Table 6:** Index Classification.

	Effective Street Width	Street Flatness	Street Cleanliness	Interface Diversity	Buffer	Shade Coverage	Green Looking Ratio	Sound Decibels
Comfort	$X_{1}\geq 3\;m$	$X_{2}=3$	$X_{3}=3$	$X_{4}>1$	$X_{5}=1$	$X_{6}\geq 90\%$	$X_{7}\geq 42\%$	$X_{8}\leq 45\;\mathrm{dB}$
General	$1.5\;m\leq X_{1}<3\;m$	$X_{2}=2$	$X_{3}=2$	$0<X_{4}\leq 1$	NA	$50\%\leq X_{6}<90\%$	$15\%\leq X_{7}<$42%	$45\;\mathrm{dB}<X_{8}\leq 55\;\mathrm{dB}$
Discomfort	$X_{1}<1.5\;m$	$X_{2}=1$	$X_{3}=1$	$X_{4}=0$	$X_{5}=0$	$X_{6}<50\%$	$X_{7}<$15%	$X_{8}>55\;\mathrm{dB}$

**Table 7:** Actual construction width and effective width of different street hierarchies.

	Arterial Road	Secondary Road				Branch Road		
Road Name	Jintang Road	13th Road	6th Road	8th Road	15th Road	N15th Road	14th Road	7th Road
Actual width	3.5 m	2.5 m	4 m	3 m	3 m	3 m	2.5 m	3 m
Average effective width	2.36 m	1.49 m	2 m	2.18 m	1.88 m	1.8 m	1.13 m	1.75 m

**Table 8:** Scores of street cleanliness.

	Service Street			Traffic Street			Landscape Street		
Street Name	6th Road	13th Road	7th Road	14th Road	N15th Road	15th Road I	15th Road II	8th Road	Jintang Road
Street flatness	2.64	2.54	2.5	2.6	3	2.4	2.64	2.67	2.71
Average	2.59			2.63			2.67		

**Table 9:** Scores of interface diversity.

	Service Street			Traffic Street			Landscape Street		
Street Name	6th Road	13th Road	7th Road	14th Road	N15th Road	15th Road I	15th Road II	8th Road	Jintang Road
Interface diversity	0.94	1	1.13	0.27	0.94	0.56	0.90	0.93	0.75
Average	0.97			0.72			0.83		

**Table 10:** The comprehensive score of streets.

	Branch Road		Arterial	Secondary Road			Branch Road		
Street Name	N15th Road	15th Road I	Jintang Road	6th Road	13th Road	8th Road	14th Road	7th Road	15th Road II
Street trees	Some parts	Some parts	Some parts	Yes	Yes	Yes	Yes	Yes	Yes
Shade coverage	32.65%	38.21%	49.78%	47.64%	63.52%	78.2%	81.36%	91.40%	93.70%

**Table 11:** The comprehensive score of streets.

	Service Street			Traffic Street			Landscape Street		
Street Name	6th Road	13th Road	7th Road	14th Road	N15th Road	15th Road I	15th Road II	8th Road	Jintang Road
Green Looking	29.04%	37.19%	33.45%	31.82%	32.05%	29.61%	44.89%	43.66%	42.73%
Average	33.12%			31.73%			43.74%		

**Table 12:** The comprehensive score of streets.

Num.	Street Name	Street Hierarchy	Street Type	Score
1	6th Lat Road	Secondary road	Service street	52.25
2	13th Long Road	Secondary road	Service street	53.93
3	14th Long Road	Branch road	Traffic street	44.95
4	7th Lat Road	Branch road	Traffic street	61.38
5	North 15th Long Road	Branch road	Traffic street	51.84
6	15th Long Road (east)	Branch road	Traffic street	39.73
7	15th Long Road (west)	Branch road	Landscape street	60.27
8	8th Lat Road	Secondary road	Landscape street	71.78
9	Jintang Road	Arterial road	Landscape street	57.48

**Table 13:** The average score of streets.

Street Hierarchy	Average Score	Street Type	Average Score
Arterial road	57.48	Service street	50.38
Secondary road	59.32	Traffic street	50.98
Branch road	51.63	Landscape street	63.18

## Data Availability

The data presented in this study are available on request from the corresponding author. The data are not publicly available due to confidentiality.
